# Clues on the Three-Vessel View for Fetal Diagnosis of Supracardiac Total Anomalous Pulmonary Venous Connection

**DOI:** 10.1016/j.case.2025.04.005

**Published:** 2025-06-19

**Authors:** Nadezhda Dzhelepova, Caroline B. Jones

**Affiliations:** a Saint Mary’s Hospital, Manchester Foundation Trust, Manchester, United Kingdom; b Royal Manchester Children’s Hospital, Manchester Foundation Trust, Manchester, United Kingdom; c Saint Mary’s Hospital, Alder Hey Children’s Foundation Trust, Liverpool, United Kingdom

**Keywords:** Supracardiac total anomalous pulmonary venous connection, Total anomalous pulmonary venous connection, Atrioventricular septal defect, Three-vessel view, Superior vena cava

## Abstract

• TAPVC is a very rare diagnosis in the settings of situs solitus and AVSD.
• The 3VV with color flow Doppler is helpful in making the diagnosis prenatally.
• Vena cava size discrepancy and large brachiocephalic vein suggest supracardiac TAPVC.

## Graphical abstract

**Figure:**
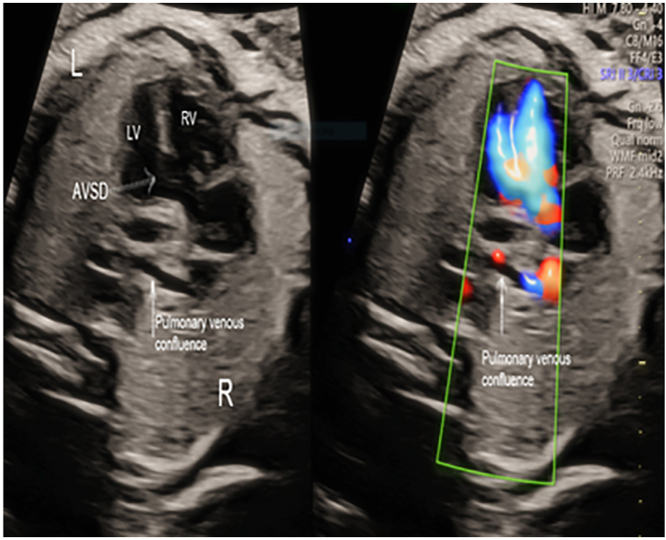

## Introduction

Total anomalous pulmonary venous connection (TAPVC) is a cyanotic congenital heart disease that has a very low antenatal diagnostic rate. TAPVC in most cases with usual situs is an isolated entity. It is extremely rare to encounter it with usual organ arrangements in combination with other major heart abnormalities, and there are just a few case reports published in the literature.

We present the case of a fetus with a rare combination of atrioventricular septal defect (AVSD) and supracardiac TAPVCs in the setting of normal visceral situs.

Initial diagnosis at 21 weeks’ gestation was of AVSD and persistent left superior vena cava (LSVC) draining to the coronary sinus, but after comprehensive review of the images, important clues to supracardiac TAPVC diagnosis were identified, and fetal echocardiography at 28 weeks confirmed the diagnosis AVSD and supracardiac TAPVC.

## Case Presentation

**Figure 1:**
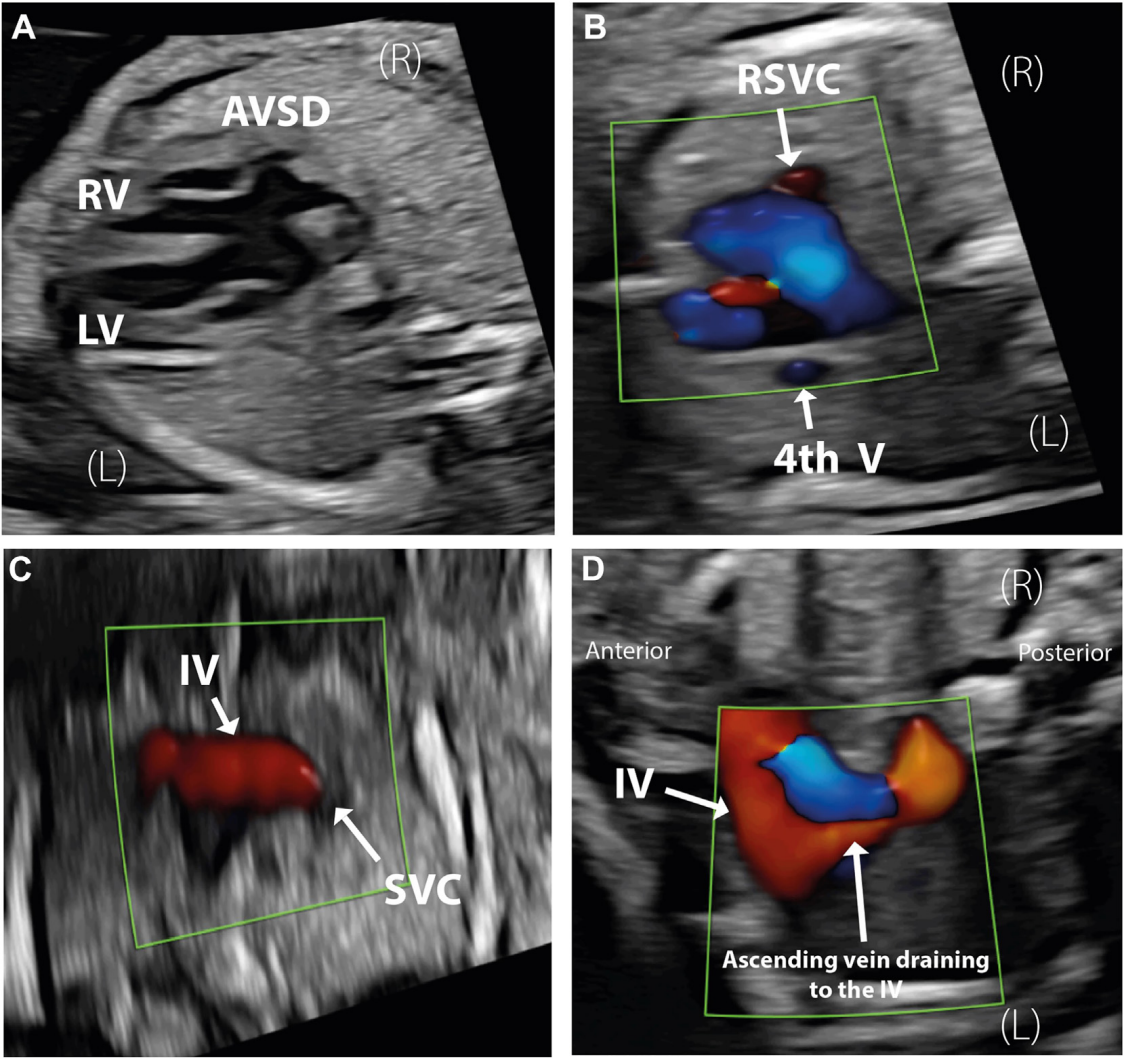
Two-dimensional fetal echocardiogram, obtained at 21 weeks of gestation, transverse plane, four-chamber view **(A)**, demonstrates levocardia, two balanced ventricles, and a common atrioventricular valve consistent with an AVSD; transverse plane, three-vessel view superior sweep with color flow Doppler **(B)** demonstrates four vessels, different color displays of the two venous vessels (opposite direction of flow), a prominent IV **(C)** with an ascending vein to the left of the PA draining to the IV **(D)**. *4th V*, Fourth vessel; *LV*, left ventricle; *RV*, right ventricle; *SVC*, superior vena cava.

A 21-year-old primigravida was referred for fetal echocardiography at 21 weeks of gestation in view of an abnormal four-chamber view. Fetal echocardiography demonstrated normal situs with levocardia and AVSD with a large primum component and a large ventricular septal component (Figure 1A, Videos 1 and 2). The three-vessel view demonstrated four vessels with, an additional vessel to the left of the pulmonary artery (PA; Figure 1B, Video 3).

The prenatal diagnosis after initial echocardiography was complete AVSD, right superior vena cava (RSVC), and persistent LSVC draining to the coronary sinus. Counseling encompassed the possibility of underlying genetic or chromosomal disorder, medical management, surgical options, and expected long-term outcomes.

Postconsultation review of images drew our attention as we noticed clues that the fourth vessel to the left of the PA was not an LSVC but an ascending vein.

There are a number of diagnostic clues available on Doppler echocardiography that suggest a diagnosis of TAPVC. These include the following:

• The two vessels on either side of the great arteries have different color flow Doppler displays, consistent with an ascending vein receiving pulmonary venous flow returning through an innominate vein (IV) and an RSVC with flow in the opposite direction (Figure 1B and D, Videos 3 and 4).
• The IV is enlarged, as it receives pulmonary venous flow (Figures 1C and D, Videos 3 and 4).

In the presence of bilateral superior vena cava, an intercommunicating bridging vein is seen only in about 30% of cases and is usually small.^1^^,^^2^

**Figure 2:**
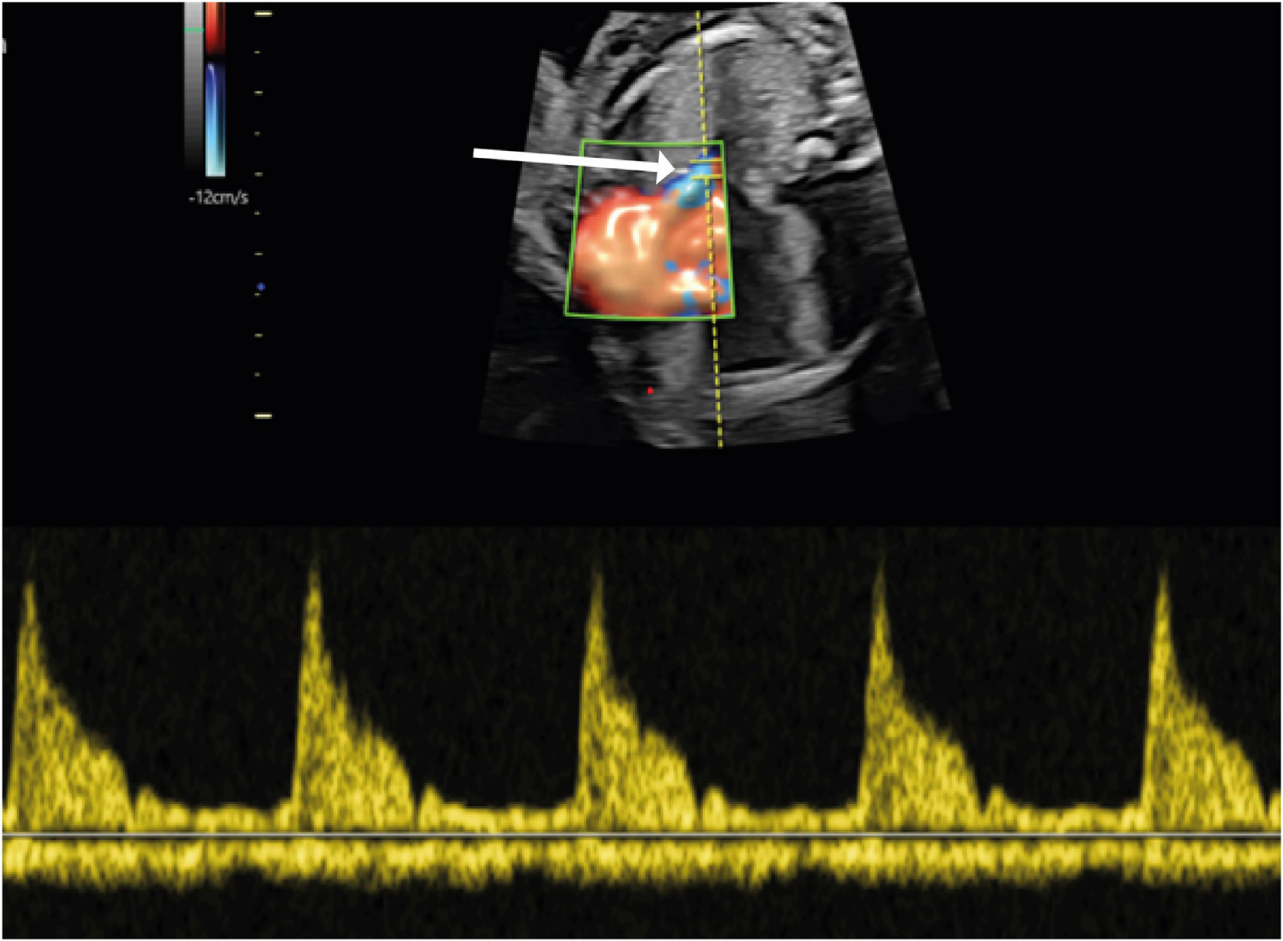
Two-dimensional fetal echocardiogram with color flow and pulsed-wave Doppler obtained at 21 weeks of gestation, transverse plane, demonstrates the spectral Doppler display from one presumed pulmonary vein (*arrow*) with an unclear site of drainage; the spectral Doppler profile is abnormal, as the pattern below the baseline does not demonstrate conventional phasic flow patterns seen with pulmonary veins.

• Color flow and pulsed-wave Doppler interrogation of the pulmonary vein flow demonstrate abnormal patterns without visualized entry into the left atrium (Figure 2)

**Figure 3:**
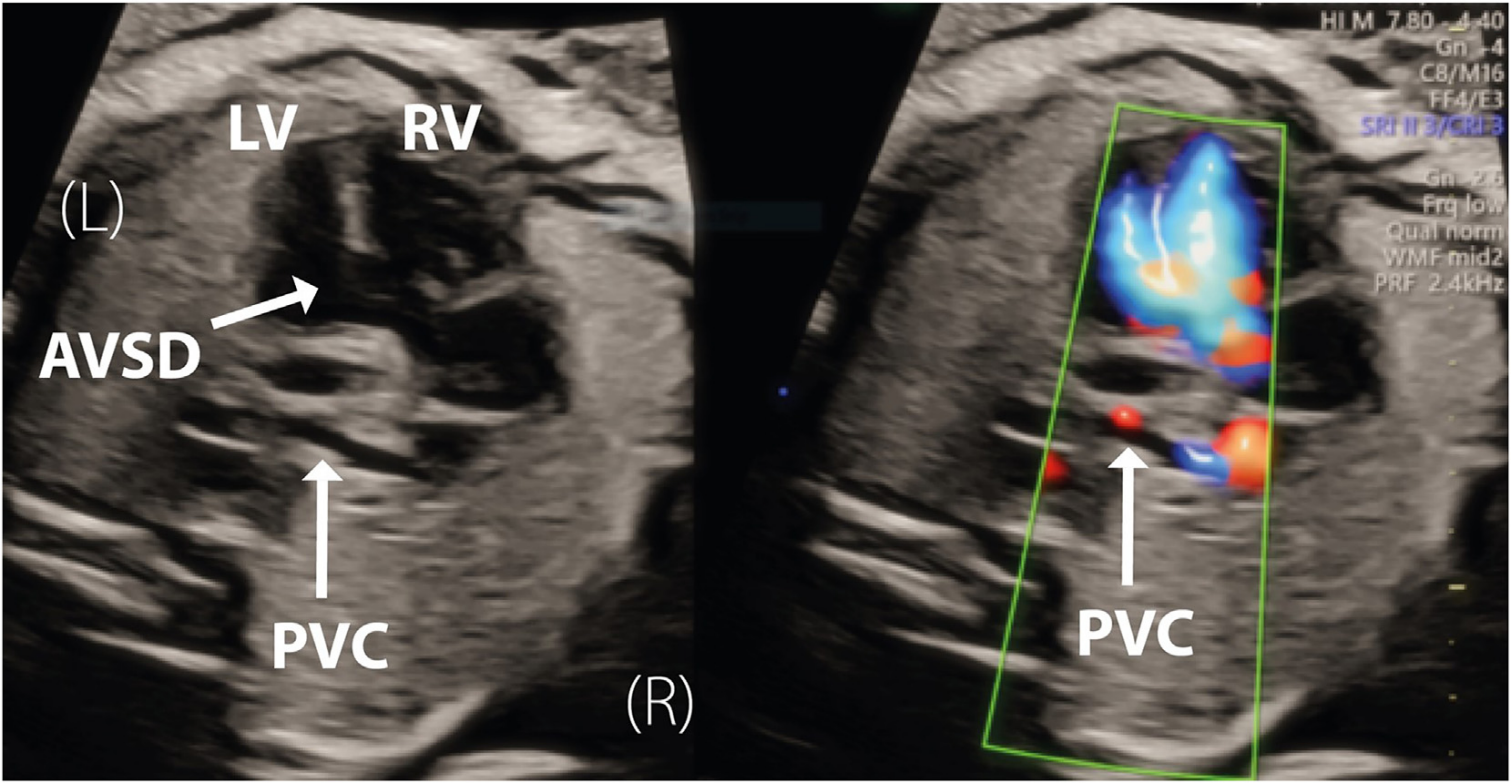
Two-dimensional fetal echocardiogram, obtained at 28 weeks of gestation, transverse plane, four-chamber view without (*left*) and with (*right*) simultaneous color flow Doppler, demonstrates AVSD (*arrow*) and, by tilting posteriorly, reveals a small pulmonary venous confluence (PVC; *arrow*) behind the left atrium. *LV*, Left ventricle; *RV*, right ventricle.

**Figure 4:**
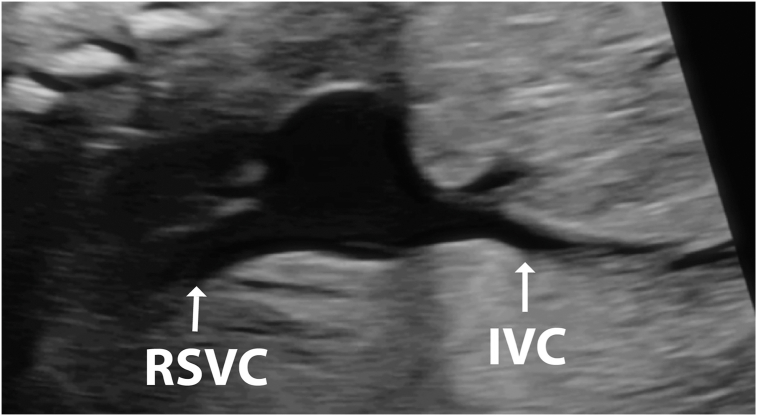
Two-dimensional fetal echocardiogram, obtained at 28 weeks of gestation, sagittal plane, bicaval view, demonstrates that the superior vena cava is larger than the inferior vena cava, which suggests drainage of the TAPVC to the RSVC.

**Figure 5:**
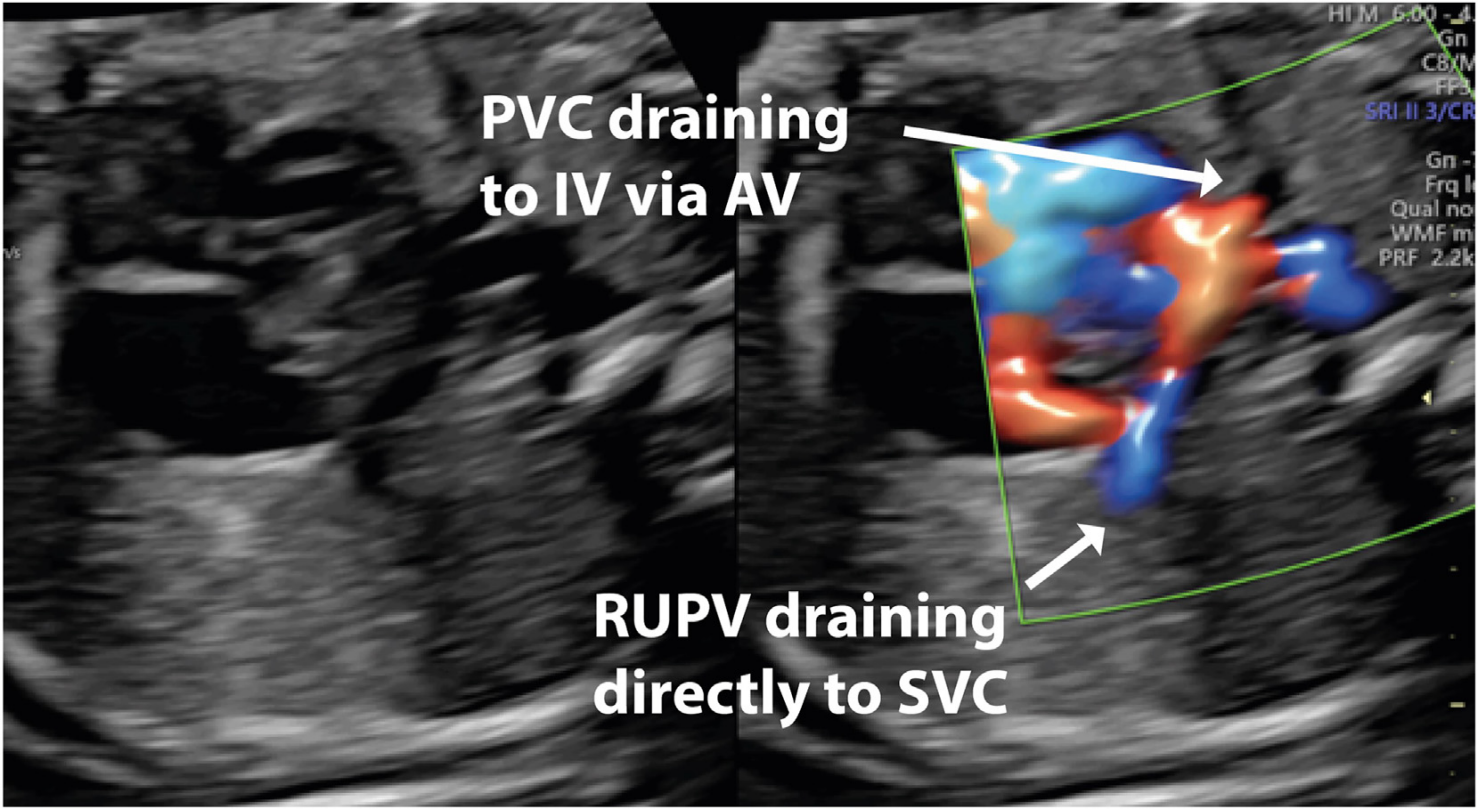
Two-dimensional fetal echocardiogram, obtained at 28 weeks of gestation, sagittal plane, without (*left*) and with (*right*) simultaneous color flow Doppler, demonstrates pulmonary veins joining the confluence, the ascending vein (AV) draining toward the IV, and the right upper pulmonary vein (RUPV) draining directly to the RSVC. *PVC*, Pulmonary venous connection; *SVC*, superior vena cava.

Fetal echocardiography at 28 weeks confirmed the diagnosis of AVSD. Tilting posteriorly, a small pulmonary venous confluence was revealed behind the left atrium (Figure 3, Video 5). The sagittal view revealed that the superior vena cava was larger in comparison with the inferior vena cava, and the ascending vein was seen draining to the IV (Figures 4 and 5). Further counseling incorporated the need for earlier surgery to reroute the pulmonary veins in the first weeks of life, with later AVSD repair.

**Figure 6:**
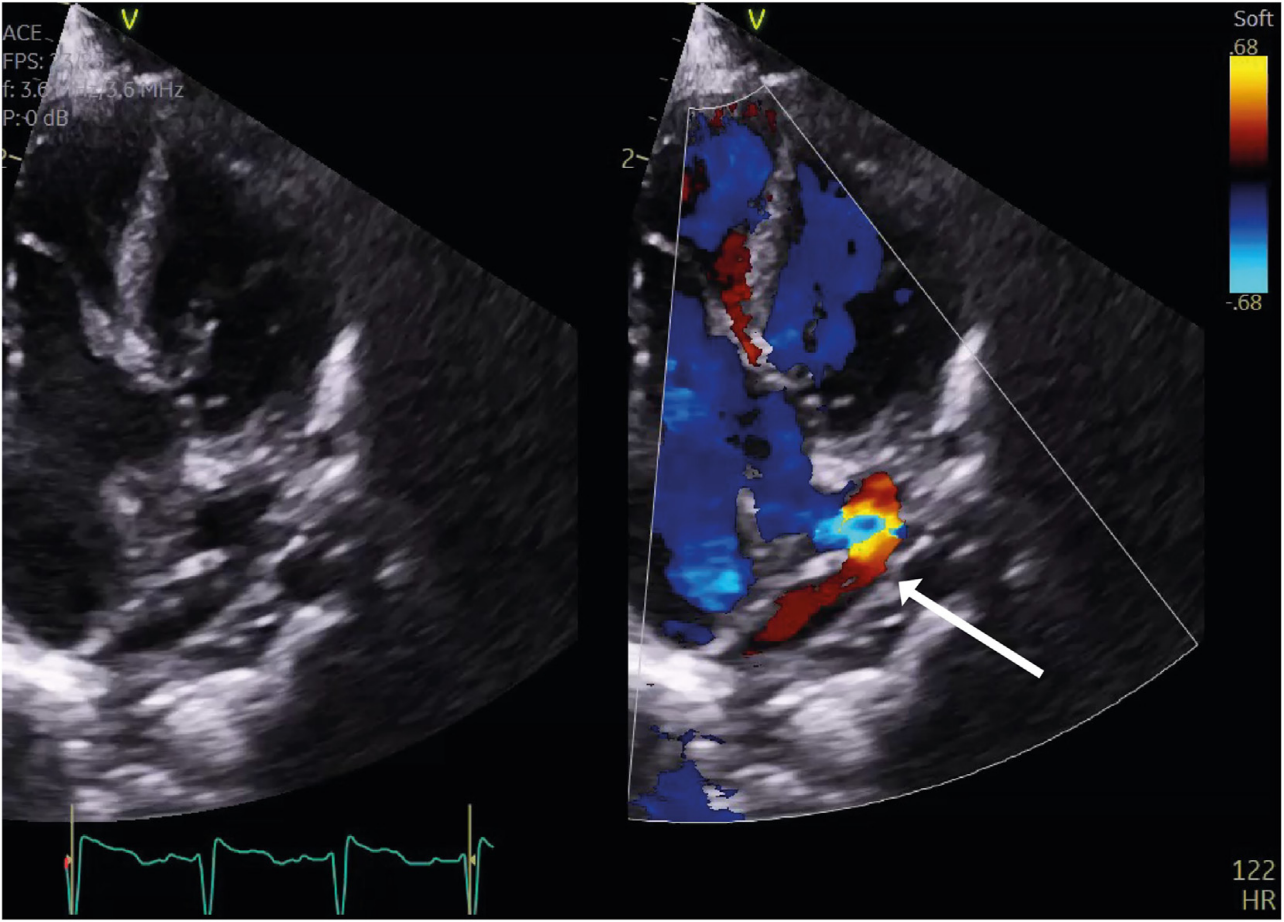
Two-dimensional transthoracic echocardiogram, obtained 2 months after TAPVC repair and PA band, apical four-chamber end-diastolic view without (*left*) and with (*right*) simultaneous color flow Doppler, demonstrates narrowing of the confluence anastomosis.

The baby was born in good condition at term, and genetic testing revealed a normal microarray. Postnatal echocardiography and computed tomography showed the usual arrangement of internal organs within the body (situs solitus), complete AVSD, and supracardiac TAPVC with one pulmonary vein draining directly to the RSVC and the remaining pulmonary veins to the confluence, with the unobstructed drainage through an ascending vein to the IV. The infant remained well, and TAPVC repair and PA band placement were performed at the age of 6 weeks (Figure 6, Video 6). The patient underwent full repair with PA debanding, AVSD repair, and revision of the pulmonary venous connection at the age of 13 months, with a very good result.

## Discussion

Our case of a fetus with normal visceral situs, supracardiac TAPVC, and complete AVSD is one of the few described in the literature^3^ and possibly the first one detected antenatally.

TAPVC is a rare heart anomaly representing 1.0% to 1.5% of congenital heart defects.^4^ It is characterized by all four pulmonary veins draining to the systemic circulation of the heart instead of the left atrium. It is classified according to the point of connection into the circulation as supracardiac, infracardiac, intracardiac, or mixed type. TAPVC typically presents early with neonatal cyanosis and may cause profound metabolic acidosis and heart failure if the pathway of return to the heart is obstructed.

Typically, in infants with normal visceral situs, TAPVC is an isolated lesion. In an international population-based study of 422 patients with TAPVC usual situs, only 26 had major congenital heart malformations (6%), and two of them had AVSD.^4^

Prenatal diagnosis of TAPVC is notoriously difficult at screening because of the low pulmonary flow in the fetal circulation,^5^ and most patients present in the early neonatal period.^5^ Different signs are suggested to help the sonographers recognize this rare entity.^6^^,^^7^ Echocardiography may reveal right ventricular dilation in the four-chamber view. Other subtle signs related to the left atrium are small size of the left atrium, increased distance between the descending aorta and left atrium, and smooth surface of the left atrium. Inspecting the area behind the left atrium, a small echo-free space might be visualized.^6^, ^7^, ^8^

Considering normal visceral situs and AVSD in our case influenced us to concentrate on assessment of common associated findings, such as ventricular imbalance, atrioventricular valve leak, outflow tract obstruction, and arch adequacy.^9^^,^^10^ A fourth vessel was seen in the three-vessel view, but we presumed that this was the LSVC, which is a variant of systemic drainage. After careful analyses of the images, we found important clues suggesting supracardiac TAPVC from the three-vessel view, even though we had not demonstrated a confluence behind the left atrium, and we could not see the small left atrium typical of TAPVC, as there was a large atrial communication.

Given the rare nature of this combination, the TAPVC was not evaluated initially, but important clues in the three-vessel view helped us distinguish an ascending vein from an LSVC and make an accurate diagnosis. This demonstrates the importance of recognizing and interpreting subtle signs when performing fetal echocardiography.

## Conclusions

We present a very rare case of AVSD and TAPVC, usual solitus situs, diagnosed antenatally. In cases of TAPVC when the pulmonary venous confluence is not demonstrated because of the specificity of pulmonary fetal circulation or the trap of expectations (extremely rare combination of AVSD, solitus situs, and TAPVC), detecting a venous vessel with color flow Doppler in the opposite direction surrounding venous vessels can help establish the accurate diagnosis.

## Ethics Statement

The authors declare that the work described has been carried out in accordance with The Code of Ethics of the World Medical Association (Declaration of Helsinki) for experiments involving humans.

## Consent Statement

Complete written informed consent was obtained from the patient (or appropriate parent, guardian, or power of attorney) for the publication of this study and accompanying images.

## Funding Statement

The authors declare that this report did not receive any specific grant from funding agencies in the public, commercial, or not-for-profit sectors.

## Disclosure Statement

The authors report no conflict of interest.

## Supplementary Data

Video 1 Two-dimensional fetal echocardiogram, obtained at 28 weeks of gestation, transverse plane at the level of the abdomen, demonstrates usual situs solitus and usual organ arrangements.

Video 2 Two-dimensional fetal echocardiogram, obtained at 21 weeks of gestation, transverse plane, four-chamber view, demonstrates levocardia, two balanced ventricles, and a common atrioventricular valve consistent with an AVSD.

Video 3 Two-dimensional fetal echocardiogram with color flow Doppler, obtained at 21 weeks of gestation, transverse plane, three-vessel view superior sweep, demonstrates four vessels, different color displays of the two venous vessels (opposite direction of flow), and a prominent IV.

Video 4 Two-dimensional fetal echocardiogram with color flow Doppler, obtained at 21 weeks of gestation, transverse plane, superior sweep of three-vessel view, demonstrates an ascending vein to the left of the PA draining to the IV.

Video 5 Two-dimensional fetal echocardiogram, obtained at 28 weeks of gestation, transverse plane, four-chamber view without (*left*) and with (*right*) simultaneous color flow Doppler, demonstrates AVSD and, by tilting posteriorly, reveals a small pulmonary venous confluence behind the left atrium.

Video 6 Two-dimensional transthoracic echocardiogram, obtained 2 months after TAPVC repair and PA band, apical four-chamber view without (*left*) and with (*right*) simultaneous color flow Doppler, demonstrates narrowing of the confluence anastomosis.
